# Differential Modulation of Beta-Adrenergic Receptor Signaling by Trace Amine-Associated Receptor 1 Agonists

**DOI:** 10.1371/journal.pone.0027073

**Published:** 2011-10-31

**Authors:** Gunnar Kleinau, Juliane Pratzka, Daniela Nürnberg, Annette Grüters, Dagmar Führer-Sakel, Heiko Krude, Josef Köhrle, Torsten Schöneberg, Heike Biebermann

**Affiliations:** 1 Institute of Experimental Pediatric Endocrinology, Charité-Universitätsmedizin Berlin, Berlin, Germany; 2 Institute of Experimental Endocrinology, Charité-Universitätsmedizin Berlin, Berlin, Germany; 3 Klinik für Endokrinologie, Zentrum für Innere Medizin, Universitätsklinikum Essen, Essen, Germany; 4 Institute of Biochemistry, Medical Faculty, University of Leipzig, Leipzig, Germany; The University of Kansas Medical Center, United States of America

## Abstract

Trace amine-associated receptors (TAAR) are rhodopsin-like G-protein-coupled receptors (GPCR). TAAR are involved in modulation of neuronal, cardiac and vascular functions and they are potentially linked with neurological disorders like schizophrenia and Parkinson's disease. Subtype TAAR1, the best characterized TAAR so far, is promiscuous for a wide set of ligands and is activated by trace amines tyramine (TYR), phenylethylamine (PEA), octopamine (OA), but also by thyronamines, dopamine, and psycho-active drugs. Unfortunately, effects of trace amines on signaling of the two homologous β-adrenergic receptors 1 (ADRB1) and 2 (ADRB2) have not been clarified yet in detail. We, therefore, tested TAAR1 agonists TYR, PEA and OA regarding their effects on ADRB1/2 signaling by co-stimulation studies. Surprisingly, trace amines TYR and PEA are partial allosteric antagonists at ADRB1/2, whereas OA is a partial orthosteric ADRB2-antagonist and ADRB1-agonist. To specify molecular reasons for TAAR1 ligand promiscuity and for observed differences in signaling effects on particular aminergic receptors we compared TAAR, tyramine (TAR) octopamine (OAR), ADRB1/2 and dopamine receptors at the structural level. We found especially for TAAR1 that the remarkable ligand promiscuity is likely based on high amino acid similarity in the ligand-binding region compared with further aminergic receptors. On the other hand few TAAR specific properties in the ligand-binding site might determine differences in ligand-induced effects compared to ADRB1/2. Taken together, this study points to molecular details of TAAR1-ligand promiscuity and identified specific trace amines as allosteric or orthosteric ligands of particular β-adrenergic receptor subtypes.

## Introduction

The group of trace amine-associated receptors (TAAR) [1] belongs to the rhodopsin-like family of G protein-coupled receptors (GPCRs) and is of importance for several physiological aspects such as proper cardiac and vascular functions (reviews [2], [3], [4], [5]). It has also been proposed that TAAR are involved as neuromodulators in brain [2], [6]. In accordance, TAAR are postulated to be linked with neurological disorders like bipolar disease [7], [8], schizophrenia [9], [10], depression and Parkinson's disease [11], [12]. In consequence, TAAR are potential new important therapeutic targets for several pathological situations [13], [14]. The first human member of this receptor group (TAAR5) was identified in 1998 [15], [16] and the term TAAR was introduced when TAAR1, TAAR8 and TAAR9 were discovered [17]. Three out of the nine hTAAR members are pseudogenes [18]. TAAR1 is activated by trace amines [6] such as tyramine (TYR), β-phenylethylamine (PEA) or octopamine (OA) [17], [19] and signals via the Gs protein/adenylyl cyclase system. In addition, it was reported that a thyroid hormone derivative, 3-thyronamine (T_1_AM) [20], [21], [22], [23], [24], [25], [26], [27], [28], [29] activates TAAR1. Remarkable differences in efficacies of T_1_AM between hTAAR1 and rodent Taar1 were observed [30]. In addition, ligands of the dopamine-, serotonine-, histamine-, or adrenergic receptors are able to induce TAAR1 mediated signaling [17], [19], [31], [32]. Surprisingly, antagonists of the serotonin receptor like cyproheptadine as well as antagonists of adrenergic receptors like phentolamine are Taar1 agonists [19]. Besides trace amines and biogenic amines also volatile amines activate human TAAR1 and murine Taar 3, 5, and 7 [33], characterizing these TAAR additionally as odorant receptors [34], [35], [36]. Finally, TAAR1 responds to psycho-active drugs [19], [37]. This points, altogether, to an enormous TAAR1 ligand-binding promiscuity that might reflect also the evolutionary link between TAAR and homologous vertebrate aminergic receptors [2], [17], [18], [38], [39], [40] or invertebrate tyramine receptors (TAR) and octopamine receptors (OAR).

Trace amines in mammalians are suggested to function as endogenous neuromodulators of classical monoamine neurotransmitters [41], [42]. In contrast, in the tyramine/octopamine system in invertebrates, the homologue to the mammalian adrenergic system [43], [44], trace amines are acting as direct neurotransmitters. Trace amines and their invertebrate receptors are involved in regulation of metabolism and of sensory and behavioral functions [44]. Several tyramine and octopamine receptors were identified in invertebrates like insects [44], [45], [46], [47] or mollusks [48]. Of note, the overlap in homologous receptor-ligand systems has also unexpected consequences. For example TAR and OAR are targets for insecticid development [44] and these substances could potentially affect TAAR or other aminergic receptors. In reverse, β-blockers have an endocrine-disrupting potential on organisms with TAR and OAR expression [49]. It is well known that particular ligands interact with several different aminergic receptors or modulate different physiological systems. Octopamine has been shown previously to be an agonist at the α-adrenergic receptor [50], [51] and the β3-adrenergic receptor [52]. Substance PEA may act as an α-adrenergic receptor antagonist [53]. The OAR of *Lymnaea stagnalis* was found to be activated by α2-adrenergic receptor ligands, which leads in case of OAR to activation of both Gs- and Gq-mediated pathways [48]. Furthermore, it can be postulated from several studies that TAAR1 function might be related with the dopamine-2 receptor [54], [55], [56], [57], [58] as well as with the serotonin receptor 5-HT(1A) [59]. Recently published evidence points to a physiological role for T_1_AM as an endogenous adrenergic-blocking neuromodulator in the central noradrenergic system [22]. In conclusion, a wide spectrum of potential ligand-aminergic receptor combinations or modulation of different physiological systems by specific ligands has been recognized. But, reflecting possible cross-combinations of the huge number of potential interaction partners this complex system is only recognized fragmentarily.

Herein we tested particular trace amines acting as agonists on hTAAR1 regarding their direct effects on hADRB1 and hADRB2 signaling. We found allosteric and orthosteric antagonistic effects of particular trace amines on ADRB1 and ADRB2. Octopamine induced different signaling effects on ADRB1 and ADRB2. Based on these findings we investigated the structural basis of TAAR ligand-promiscuity by comparative studies to other aminergic receptors and found similarities and differences between aminergic receptors which help to explain differential modification of signaling induced by trace amines.

## Results

### Different effects of trace amines at hTAAR1 and human β-adrenergic receptors

**Figure 1:**
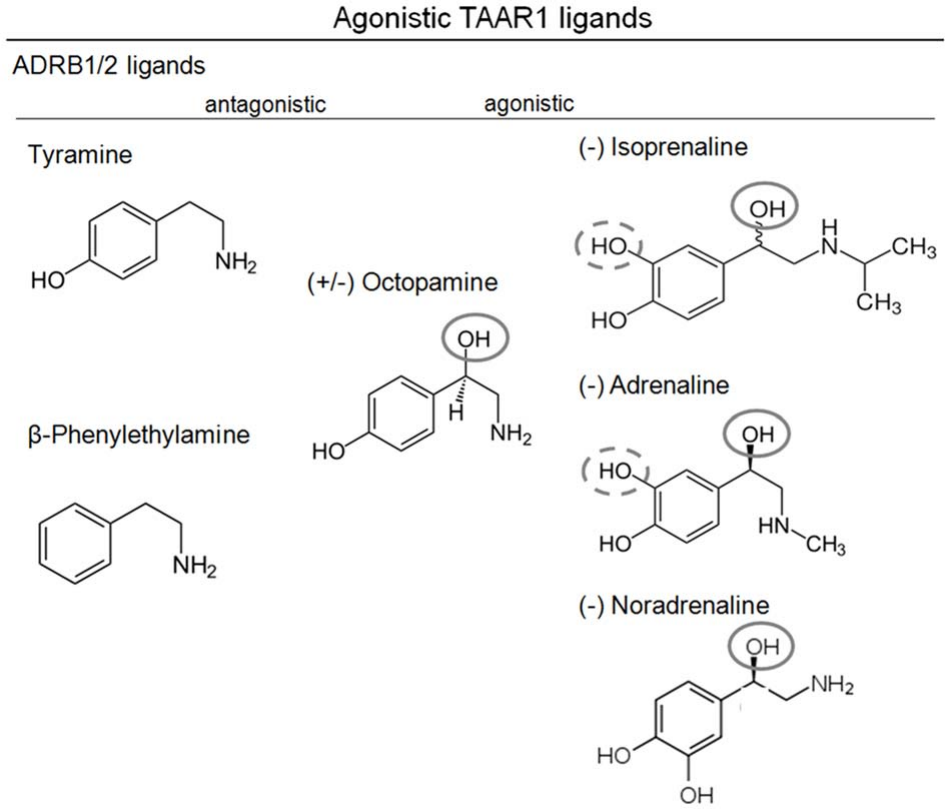
Molecular structures of ADRB1/2 and TAAR1 ligands. The trace amines TYR, PEA and OA are agonists of TAAR1. In contrast, TYR and PEA are antagonists on ADRB1/2, most likely due to less hydroxyl groups compared OA or already known agonistic beta-adrenergic ligands (grey circles). OA has an additional hydroxyl group (grey circle), but yet a hydroxyl group less (dashed grey circle) compared to full ADRB1/2 agonists isoprenaline, adrenaline and noradrenaline.

**Figure 2:**
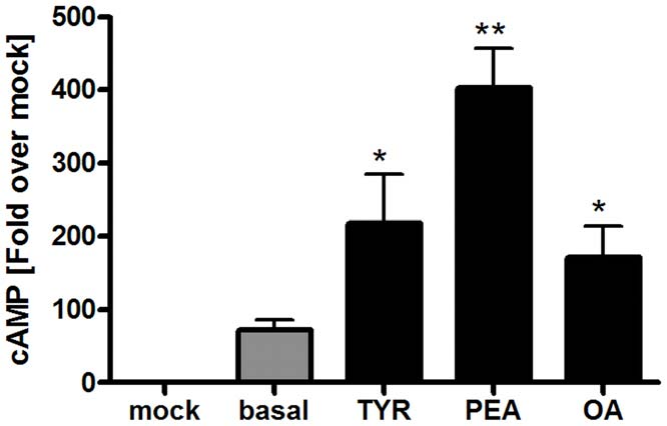
Functional characterization of hTAAR1 interacting with different trace amines. HEK293 cells transiently transfected with hTAAR1 and were incubated for 45 minutes with 10 mM of PEA, OA and TYR. Basal signaling activity as well as activation of the Gs protein/adenylyl cyclase pathway was determined by AlphaScreen technology. Data represent mean ± SEM of cAMP accumulation after stimulation from 4–5 independent experiments performed in triplicates. TAAR1 showed an elevated ligand independent basal activity. PEA was the most potent agonist (p<0.01), followed by TYR (p<0.05) and OA (p<0.05). Data were analyzed using a paired one-tailed t-test.

Ligands PEA, TYR and OA (figure 1) activate hTAAR1 [17], [19] with subsequent stimulation of adenylyl cyclase and cAMP formation (figure 2, supplemental figure S1). Of note, hTAAR1 exhibits a high level of basal constitutive (ligand independent) signaling activity (73±14 nM cAMP) compared to mock transfected cells (figure 2). This hTAAR1 characteristic is in accordance to the reported effects of inverse agonists effecting ligand independent signaling activity of TAAR1 [60], [61].

For describing the functional effects of PEA, TYR and OA we use the following terms: allosteric antagonist if a non-competitive effect on ISOP is observed and orthosteric antagonist if a competitive effect occurs. The most potent agonist tested was PEA (Emax = 403±54 nM), followed by TYR (Emax = 219±67 nM) and OA (Emax = 172±42 nM) (figure 2 and supplemental figure S1).

**Figure 3:**
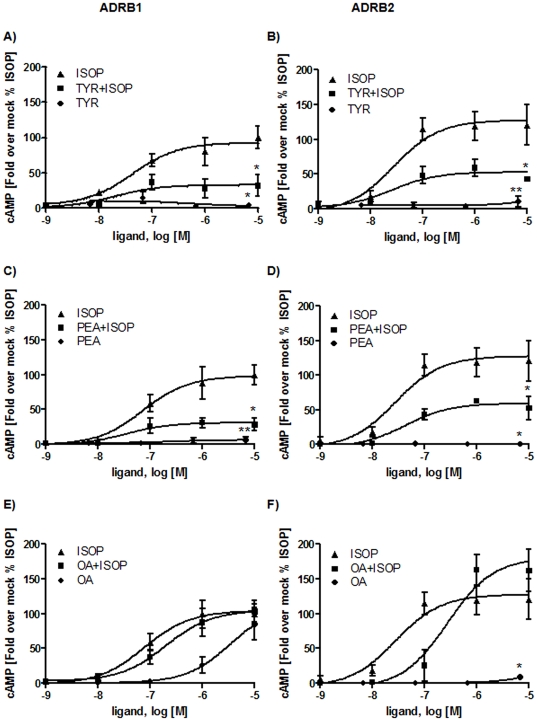
Characterization of hADRB1 and hADRB2 after trace amine challenge. HEK293 cells were transiently transfected with hADRB1 and hADRB2, respectively. Cells were pre-incubated with increasing concentrations of the trace amines TYR, PEA or OA (6.7 nM to 6700 nM) for 15 minutes. For competition studies ISOP with increasing concentrations (10000 nM to 1 nM) was added and incubated for additional 30 minutes. As controls hADRB1 and hADRB2 were incubated with each substance alone with the same concentration for 45 minutes. Three to four independent experiments measured in duplex or tripletts mean ± SEM are depicted here. Fold over mock was calculated and expressed as percentage of ISOP stimulation (100%). Maximal values were statistically analyzed using paired one-tail t-test compared to ISOP maximal stimulation. **A-B**) hADRB1 and hADRB2 were stimulated with ISOP and TYR alone and with both substances simultaneously. TYR shows no agonistic effect on hADRB1 (p<0.05) and hADRB2 (p<0.01), but acts as an allosteric antagonist on hADRB1/2, indicated by a decreased maximum of stimulation (p<0.05) with comparable EC_50_ values. **C-D**) PEA shows no agonistic effects on both hADRB1 (p<0.01) and hADRB2 (p<0.05) but leads to a decreased maximum of stimulation by ISOP when pre-incubated with PEA (p<0.05). The EC_50_ value is similar to stimulation with ISOP alone. **E**) OA acts as an agonist on hADRB1 but with a decreased efficacy. Simultaneous incubation with ISOP and OA reveals no antagonistic effect of OA on hADRB1 (table 1). **F**) OA is an orthosteric antagonist on hADRB2, indicated by a right shift of the EC_50_ value. OA however showed no agonistic effect on hADRB2 (p<0.05).

Isoprenaline (ISOP) is a full agonist to β-adrenergic receptors and was also reported as an agonist to TAAR1 [19]. We functionally tested the effects of particular trace amines at the human ADRB1 and ADRB2 (figure 3). Therefore, HEK293 cells (ATCC-LGC, Wesel, Germany) were transiently transfected with hADRB1 or hADRB2 and incubated with TYR, PEA or OA alone or in presence of ISOP. For competition experiments we prestimulated ADRB1 and ADRB2 with each trace amine TYR, PEA and OA in concentrations ranging from 6.7 nM to 6700 nM [17], [19]. Then, ISOP in concentrations from 1 nM up to 10000 nM was added and cAMP accumulation was measured. Controls were stimulated with each trace amine and ISOP alone, respectively.

As shown in figure 3A-B, TYR acted as an allosteric antagonist at both, ADRB1 and ADRB2, by decreasing the E_max_ values to 30% and 60% of the wild type receptor, respectively, when compared to ISOP alone. A small increase of ADRB2 signaling at highest TYR concentrations was not significant. The concentration-response curves revealed no shift in the EC_50_ value (figure 3A, table 1) indicating a non-competitive allosteric effect of TYR on ISOP-stimulated ADRB1 and ADRB2. Similarly, PEA non-competitively antagonized signaling of both ADRB1 and ADRB2 (figure 3C, D).

**Table 1:** EC_50_ values of different agonists at hTAAR1, hADRB1 and hADRB2.

Receptor	Ligand	EC_50_ [nM][nM]	Emax [nM]
ADRB1	ISOP	61±10	270±47
	TYR	-	*
	PEA	-	*
	OA	3129±461	217±30
	TYR+ISOP	39±22	82±15
	PEA+ISOP	41±9	87±8
	OA+ISOP	210±74	270±104
ADRB2	ISOP	29±5	328±44
	TYR	-	*
	PEA	-	*
	OA	-	*
	TYR+ISOP	20±3	167±35
	PEA+ISOP	44±9	162±52
	OA+ISOP	249±70	443±50
hTAAR1	TYR	1540±251	219±67
	PEA	260±16	403±54
	OA	4170±1470	172±42

EC_50_ values were calculated using GraphPadPrism from concentration response curves of measured cAMP accumulation. Displayed is the mean ± SEM from n≥3 independent experiments. Emax values mean ± SEM from n≥3 independent experiments were calculated from fold over mock data. “–“not determinable with sufficient accuracy, “*” not determinable due to extremely low stimulation.

In contrast, OA acted as an agonist to hADRB1 (figure 3E), but with lower potency (EC_50_ 3129±461 nM) than ISOP (EC_50_ = 61±10 nM) (table 1). OA together with ISOP showed slightly differences in their EC_50_ values. Nevertheless, there were no changes in E_max_ values (table 1, figure 3E) detectable. Interestingly, OA did not activate ADRB2 but acted as an orthosteric antagonist by inhibiting ISOP activation competitively (figure 3F).

### Structural similarities and differences between hTAAR and homologous aminergic receptors

**Figure 4:**
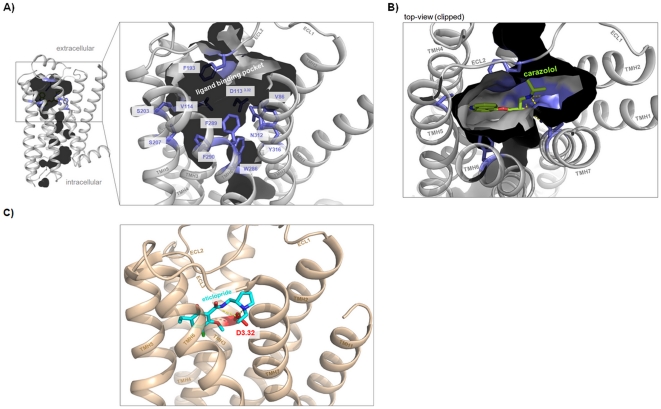
Defining the ligand binding region of aminergic receptors. **A**) The pocket-like ligand binding region (inner crevice surface) of the human β2-adrenergic receptor (pdb entry code 2RH1) is surrounded by amino acids (lilac sticks, labeled) which are also known from mutagenesis studies to be important for ligand binding and signal transduction. **B**) The inverse agonist carazolol is embedded tightly in this pocket of the β2-adrenergic receptor (top-view) and interacts with residues of TMH 3, 5, and 7 by hydrogen bonds [62]. Differences in binding and effects on receptor conformation compared to agonists were found to be relatively small, mainly manifested in the interaction pattern to TMH5 or induced side chain rotamer conformations at TMH5 (amino acids at positions 5.41, 5.42 and 5.46) [80]. **C**) The crystal structure of dopamine-3 receptor (pdb entry code 3PBL) with the antagonist eticlopride [65] shows a similar localization between the transmembrane helices compared to carazolol in the adrenergic receptor (B). An aspartate (red stick) at position 3.32 in helix 3 is well known to function as an anchor point for binding of ligands at aminergic receptors.

**Figure 5:**
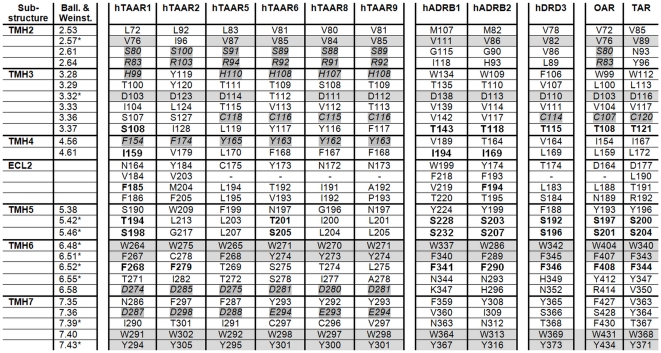
Amino acids covering the ligand binding region of β-adrenergic receptors and TAARs. Amino acids covering the ligand binding regions of β-adrenergic receptors 1 and 2 are compared with corresponding residues of human TAAR subtypes, dopamine-3 receptor and invertebrate tyramine (*Apis*) (TAR) or octopamine receptor (*Bombyx*) (OAR) (see also amino acid sequence alignment figure S2). This comparison reveals potential overlapping binding determinants which are predestinated to interact with shared ligands. The amino acids of ADRB1 and ADRB2 ligand binding region are identified by analyzing solved crystal structures complexed with different ligands (figure 4). Residues which are directly involved in ligand binding at adrenergic receptors are marked by a star-symbol (*). The numbering is provided by the *Ballesteros and Weinstein* numbering scheme [66] and consecutively to the entire amino acid sequence. Especially TAAR1 shows similar or even identical side chains (bold) with the adrenergic receptors, dopamine-3 receptor and OAR or TAR. Highly conserved amino acids between all receptors are marked by a gray background. Conserved residues within hTAAR subtypes are in italic with a partial gray background. These five residues are located at TMH 2, 3, 6 and 7 and likely encode TAAR specificities compared to other aminergic receptors.

**Figure 6:**
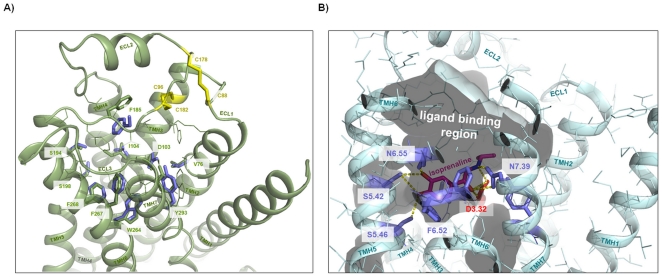
TAAR1 shows similarity in the ligand binding region compared with β-adrenergic receptors. **A**) Superimposition of the hTAAR1 homology model (extracellular top-side view, green backbone) and the hADRB2 crystal structure (lilac sticks hADRB2, backbone not shown) reveals similarities of residues which are known to be important for ligand binding and signal transduction in adrenergic receptors (figure 4 and figure 5). These identical amino acids (sticks) should be involved in binding of shared ligands like isoprenaline. Additional side chains of hTAAR1 covering the putative ligand binding region (figure 5) are represented as green lines. Cysteine bridges (yellow) between loops 1 and 2 or loop 2 and TMH3 are highlighted and labeled. **B**) The recently published crystal structure of the turkey β1-adrenergic receptor co-crystallized with the agonist isoprenaline (pdb entry code 2Y03 [80]) shows main key players for intermolecular hydrogen bonding (yellow dotted lines) at aminergic receptors like side chains at positions D3.32 (TMH3) (red stick); S5.42, S5.46 (TMH5); and N7.39 (TMH7) (lilac sticks). Interestingly, the inner-pocket surface (translucent) between the extracellular ends of the helices and ECL2 shows an unoccupied volume which might form a second binding site for small molecules.

Our knowledge concerning ligand binding at β-adrenergic receptors and other members of the rhodopsin-like GPCR family has been dramatically increased since crystal structures of ADRB1, ADRB2, and dopamine-3 receptor (DRD3) in complex with antagonists or agonists recently became available [62], [63], [64], [65]. These crystal structures provide molecular details of intermolecular interaction between receptor proteins and small molecules with respect to the location of ligands, their orientation and intermolecular interaction partners (figure 4A–C). TAAR, OAR/TAR of invertebrates and classical aminergic receptors show high amino acid sequence identities (supplemental material figure S2). To compare hTAAR with aminergic receptors we analyzed determinants of the β2-adrenergic ligand-binding region (figure 4 and figure 5) and designed human TAAR homology models based on the β2-adrenergic receptor conformation (figure 6A, supplemental material figure S3). This is reasonable because TAAR1 shows highest sequence similarity to ADRB1 (∼35%) and ADRB2 (∼39%) and TAAR1 is activated by adrenergic receptor agonists such as isoprenaline [19], [30].

#### Structural properties and specificities of TAAR

TAAR are structurally constituted by an extracellular N-terminal tail (Ntt), seven transmembrane helices (TMH1-7), three extracellular loops (ECL1-3), three intracellular loops (ICL1-3) and an intracellular C-terminal part (Ctt). TAAR share specific highly conserved amino acids in the TMH with all rhodopsin-like GPCR [66]. One of the main structural difference between GPCR is the conformation and spatial localization of ECL2 [67]. While in rhodopsin a β-sheet like fold is observable, crystal structures of adrenergic receptors show a helical ECL2 conformation. We suggest based on the sequence alignment that ECL2 of TAAR is in an adrenergic-receptor-like helical conformation, fixed by two cysteine-bridges: i. the highly conserved cysteine bridge between ECL2 and TMH3, and ii. between ECL2 and ECL1 (figure 6A). Disulphide-bridged extracellular loops 1 and 2 are also observable in the crystal structure of adenosine-2A receptor (pdb entry code 3EML [68]) and this interaction is of structural and functional relevance [69], [70].

#### Defining the ligand binding region of aminergic receptors

As shown in figure 4 the ligand binding region of adrenergic receptors is located between the extracellular ends of the transmembrane helices. This pocket-like crevice is covered by specific amino acids [71] which are predestinated for interaction with ligands in different particular modes [72]. For comparison of amino acids (figure 5) which are potentially important for ligand binding at different aminergic receptors we depicted those amino acids of ADRB2 that are determinants of this spatial region (figure 4, figure 6A). We found that most of the ligand binding-sensitive side chains in aminergic receptors (e.g. positions 2.57, 3.32, 6.48, 6.51, 7.53) are also conserved in TAAR. This is also reflected by our homology model of hTAAR1 compared with the crystal structure of ADRB1 (figure 6A). hTAAR1 is, in contrast to other TAAR, characterized by six additional residues which are similar to the binding pocket of β-adrenergic receptors (figure 5), including specific amino acids at TMH5 positions 5.42 and 5.46. Their side chains are known to be important for signaling and ligand specific signaling effects [73], [74]. These two residues are also conserved in DRD3 and TAR/OAR. However, the observable high sequence similarity might explain TAAR1 ligand promiscuity. In contrast, also significant differences between ADRB1/ADRB2 compared to hTAAR1 are observable by comparison of amino acids in the ligand binding region. Especially the asparagines at positions 6.55 (TMH6) and 7.39 (TMH7) are known to be important for ligand binding at adrenergic-receptors (figure 6B), but they are absent in hTAAR or OAR and TAR.

#### Comparison of hTAAR subtypes

On note, there are also molecular differences in the ligand-binding region between hTAAR1 and further hTAAR group members (figure 5). Mapping amino acids that are different between TAAR1 and other hTAAR subtypes to the structural homology models of all hTAAR members a particular spatial region between TMH3, TMH5 and TMH6 is most diverse in biophysical properties (supplemental material figure S3). Such region with divergent properties might cause differences of TAAR subtype-ligand sensitivity or induced effects of ligands [3], [18], [75], [76]. Despite these differences between hTAAR a specific set of identical amino acids in the ligand binding region of TAAR is constituted by residues S2.61, R2.64 (TMH2); H3.28, S/C3.36 (TMH3); F/Y4.56 (TMH4); D6.58 (TMH6); and D7.36 (TMH7). These amino acids with few exceptions are not found in DRD3, ADRB1/2 or TAR and OAR. In conclusion, those residues and spatial potential ligand-binding region between TMH2, TMH3, TMH6 and TMH7 (supplemental material figure S3) are predestinated to interact with so far unknown TAAR-selective ligands.

## Discussion

A diverse spectrum of ligands including trace amines interacts with aminergic receptors and modulates different physiological functions. We show here, that the ligand-binding region of TAAR1 is characterized by high similarity to other aminergic GPCR like ADRB1, DRD3 or TAR/OAR. Trace amines such as PEA, TYR and OA (figure 1) can activate hTAAR1 via the Gs protein/adenylyl cyclase pathway. Human TAAR1 possesses a ligand independent basal activity and PEA is the most potent agonist, followed by TYR and OA (figure 2, supplemental material figure S1). In accordance, a previous experimental study demonstrated that ligands on hTAAR1 interact with typical binding-motifs of aminergic receptors [77]. Of note, also for non-human TAAR3 and TAAR4 subtypes (e.g. TAAR orthologs from rat and mouse) ligand promiscuity was reported, but for different ligands compared to hTAAR1. TAAR3 and TAAR4 are pseudogenes in humans [18].

We here tested TAAR1 agonists TYR, PEA and OA regarding their effects on ADRB1/2 signaling. We found that TYR and PEA are allosteric antagonists at both tested β-adrenergic receptors, whereas OA was a weak orthosteric ADRB2-antagonist and a weak ADRB1-agonist. This finding revealed trace amines TYR, PEA and OA as potential endogenous ligands for ADRB1 and ADRB2, but primarily as partial antagonists. Secondly, despite high similarities between the hTAAR1 and ADRB1/2, allosteric modification of isoprenaline induced activation of ADRB1 and ADRB2 by TYR and PEA points to differences in details of ligand binding and action. The ligands PEA and TYR are distinguished from OA and ISOP by a lower number of hydroxyl-groups at the aliphatic chain (figure 1). The additional hydroxyl-group of OA and ISOP interacts via H-bond with an asparagine (position 7.39) at TMH7 as observed in the ADRB1 crystal structure (figure 6B). Of special note, amino acids side chains at this position were already identified being involved in determination of binding properties and selectivity of ligands on rat and mouse TAAR1 [78]. This anchor-contact towards TMH7 (H-bond to 7.39) can not be assumed for TYR and PEA due to a lack of this specific hydroxyl-group (figure 1). In consequence, differences in the orientation of TYR and PEA at ADRB1/2 compared to observed localization of ISOP in the orthosteric binding pocket of ADRB1 between TMH3, TMH5, TMH6, and TMH7 are likely. In conclusion, allosteric effects of PEA or TYR for co-stimulation with ISOP can be explained by a different orientation of both ligand subtypes in the ligand-binding region. The ADRB1 crystal structure co-crystallized with ISOP reveals a spatial region for potential binding of allosteric antagonists TYR and PEA close to the binding site of agonist ISOP (figure 6B). Based on our findings we assume a second binding site at ADRB1 and ADRB2 which can be occupied by small molecule antagonists.

OA with a hydroxyl-group at the phenyl ring and at the hydrophilic side chain (figure 1) is able to interact with N7.39 at TMH7 like ISOP at ADRB1 and is therefore likely localized similarly to other adrenergic ligands and induces orthosteric effects. Studies on invertebrate adrenergic receptor-like trace amine receptors point indeed to similar or identical details of octopamine/receptor interactions compared to adrenergic receptors. Huang and co-workers [79] provided evidence for participation of serine D3.32 (TMH3) and S5.42 (TMH5) for activation of octopamine receptor by OA. However, the different effects of OA at ADRB1 (agonistic) versus ADRB2 (neutral in the basal state and antagonistic for ISOP treatment) are likely related to peculiarity of interaction between the hydroxyl-group at the phenylic ring system with hydrophilic residues at TMH5 (positions 5.42, 5.46) as suggested for other ligands [80]. Alternatively, differences to other adrenergic-ligands might be caused by changed interactions to the asparagine at position 6.55 which is observable at ADRB1 complexed with ISOP (figure 6B). Of note, an aromatic residue at TMH6 at position Y6.55 was found as a molecular switch for G-protein preference of OAR [81].

Based on analyses of TAAR homology models and sequence comparison we showed that the general ligand binding region of hTAARs is characterized by high amino acid conservation specifically in a crevice between the interfaces of TMH 2, 3, 6 and 7 (figure 5 and supplemental figure S3). This region is termed *minor pocket* [82] as part of the entire ligand binding region known for family A GPCRs [72]) and is not occupied by isoprenaline in the ADRB1 crystal structure (figure 6B), nor by carazolol in ADRB2 (figure 4B). Therefore we here hypothesize a preference of this pocket for a shared endogenous and also so far unknown TAAR ligand(s). In addition, our comparative studies also revealed that hTAAR1 is significantly different from all other hTAAR group members in the general ligand binding region. This likely would explain non-responsiveness of other hTAARs to particular ligands of TAAR1.

Taken together, we here present molecular details causing TAAR1 ligand promiscuity. We also found different effects of trace amines at hTAAR1 versus hADRB1 and hADRB2 which can be explained by complementary properties at ligands and receptors. Particular TAAR1 agonists are inhibitors of β-adrenergic receptor subtypes. These differences in ligand-induced effects are caused by specific properties of TAAR1 compared to ADRB1 and ADRB2 in the ligand binding region. Interestingly, an antagonistic effect on β-adrenergic signaling was reported in the early 80′s for several thyronamines. T_3_AM, 3,5-T2AM, and T_0_AM interfere with ligand binding to adrenergic receptors expressed at the plasma membrane of turkey erythrocytes and inhibited the activation of cAMP synthesis [83], [84]. Of special note, octopamine acts as an orthosteric ligand for ADRB1 and is an ADRB2-antagonist. These findings are also interesting under aspects of ligand development for TAAR [3], [60], [78], [85], [86], [87], [88], [89], [90], which has to be carefully explored concerning their potential interactions to other aminergic receptors.

## Materials and Methods

### Cloning of hTAAR1 and β-adrenergic receptors

Full length human TAAR1 (hTAAR1, NM_138327.1) was subcloned into the eukaryotic expression vector pcDps, N-terminally tagged with an hemagglutinin (5′ YPYDVPDYA 3′) epitope via *Kpn*I and *Spe*I restriction sites. For better cell surface expression hTAAR1 was additionally N-terminally fused with the first 20 amino acids of the bovine Rhodopsin [18], [33]. The human β1-adrenergic receptor (hADRB1, NM_000684.2)-pcDps construct cloned via *Kpn*I/*Spe*I containing N-terminal (HA)-tag and C-terminal Flag-tag (5′ DYKDDDDK 3′).

Human β2-adrenergic receptor (ADRB2, NM_000024.5) was subcloned via *EcoR*I and SpeI with an N-terminal (HA)-tag as well. All constructs in the eukaryotic expression vector (pcDps) were sequenced for verification with BigDye-terminator sequencing (Perkin-Elmer, Weiterstadt, Germany) and an automatic sequencer (ABI 3710xl; Applied Biosystems, Foster City, CA).

### Cell culture, cAMP assay and ligand induced effects

Human embryonic kidney cells (HEK293) were cultured in Minimum Essential Medium (MEM) Earle's (Biochrom AG) supplemented with 10% FBS (PAA Laboratories GmbH), 100 U/ml penicillin, and 100 µg/ml streptomycin (Biochrom AG) and 2 mM L-glutamine (Invitrogen) at 37°C with 5% CO_2_. 48 well plates were coated with Poly-L-Lysine (Biochrom) and HEK293 cells were seeded with 37,500 cells per well. Transient transfection in triplicates with 84 ng DNA/well using metafectene according to manufactures instructions (Biontex, Munich, Germany) was performed 28 hours later. 40 hours after transfection cells were pre-incubated for 5 minutes with stimulation buffer containing of MEM Earle's media and 1 mM 3-isobutyl-1-methylxanthine (IBMX, Sigma). This stimulation buffer was used for all further steps. For ligand competition experiments cells were incubated with tyramine, 2-phenylethylamine or (±) octopamine ranging from 6.7 nM to 6700 nM, diluted in stimulation buffer for 15 minutes followed by a stimulation with (-) isoprenaline in concentrations ranging from 1 nM to 10000 nM for 30 minutes. Ligands were all purchased from Sigma, Munich. Controls were incubated with tyramine, β-phenylethylamine, octopamine or isoprenaline for 45 minutes. All reactions were performed at 37°C with 5% CO_2_ saturated air and stopped by aspirating medium. Cells were lysed at 4°C for 2 h on a shaking platform with cell lysis buffer containing 5 nM HEPES, 0.1% BSA, 0.3% Tween20 and 1 mM IBMX.

The competitive cyclic adenosine-monophosphate (cAMP) assay via Alphascreen (Perkin Elmer Life Science, Inc., Boston, MA) was carried out according to the manufacturers' protocol. Briefly, 5 µl of each sample were transferred to a 384 well plate. Acceptor beads were diluted in 1X HBSS with 1M HEPES, 0.1% BSA, pH 7.4 and incubated for 30 minutes at room temperature. Then donor beads and 50 µM biotinylated cAMP diluted in the same way were added and incubated for an hour at room temperature. Measurement of the plate was performed using Berthold Microplate Reader (Berthold Technologies GmbH & Co. KG). Results are expressed in fold over mock (unstimulated empty vector). Dose response curves and bar graphs with mean ± SEM as well as statistical analysis (paired one-tailed t-test) were generated using GraphPad Prism Version 4.03.

### Structural homology models of hTAARs

Crystal structures of inactive receptor conformations serving for GPCR homology modeling have been published for several family A GPCR members like rhodopsin, adenosine- or β-adrenergic receptors (reviewed in [67]). For modeling of the human TAARs 1, 2, 5, 6, 8, and 9 we used the inactive structural conformation of the β-2 adrenergic receptor (pdb entry 2RH1, [62]), based on high sequence similarity between hTAAR1 and β-adrenergic receptor 2 (39% similarity, Blosum62 matrix).

The amino acid sequence alignment (supplemental material figure S2) for assignment of corresponding amino acid positions was made by usage of the Hidden-Markov algorithm derived for several GPCRs in comparison to the available GPCRs structures [67]. Refinements of the loop regions were made manually.

The software package Sybyl 7.3.5 (Tripos Inc., St. Louis, Missouri, 63144, USA) was used for structural modeling approaches. Gaps of missing residues in the loops of the template structure were closed by the ‘Loop Search’ tool in Sybyl. Substituted side chains and loops of each homology model were subjected to conjugate gradient minimizations until converging at a termination gradient of 0.05 kcal/(mol*Å)) and molecular dynamics simulation (4 ns) by fixing the backbone of the transmembrane helices. The AMBER 7.0 force field was used. Finally, the models were minimized without constraints for 2ns using the AMBER 7.0 force field. Stability of the models was validated by checking the geometry using PROCHECK.

Structure images were produced using PyMOL Molecular Graphics System, version 1.3, Schrödinger, LLC. To facilitate comparison of different GPCRs we used both the amino acid numbering of the entire TAARs with their signal peptides and the Ballesteros-Weinstein numbering scheme [66].

## Supporting Information

Figure S1 **Dose response curves of hTAAR1 agonists.** The trace amines tyramine (TYR), beta-phenylethylamine (PEA) and octopamine (OA) activate hTAAR1 via Gs/adenylate cyclase signaling. HEK293 transiently expressing hTAAR1 were stimulated with each trace amine in concentrations ranging 0.1 mM to 10 nM. Shown are dose response curves fold over basal means ± SEM from n≥4 independent experiments of measured cAMP in triplicates as described in *Material and Methods*. PEA was the most potent agonist (p<0.01), followed by TYR (p<0.05) and OA (p<0.05) for 10 µM each ligand. Data were analyzed using paired one-tailed t-test tested against basal value of hTAAR1.
(TIF)

Figure S2 **Amino acid sequence alignment of hTAAR1 and homologous receptors.** The alignment compares amino acids of ADRB1, ADRB2, human TAAR, invertebrate octopamine (OAR) and tyramine (TAR) receptors and the human dopamine-3 receptor (DRD3). Particular background colors indicating conservation among different receptors and reflecting biophysical properties of the amino acid side chains: black – proline, blue – positively charged, cyan/green – aromatic and hydrophobic, green – hydrophobic, red – negatively charged, gray – hydrophilic, dark-red – cysteines, magenta – histidine. The putative helix dimensions and loop regions are assigned according to observable features in the crystal structure of the inactivated β2-adrenergic receptor (pdb entry code 2RH1). Furthermore, in homology to the ligand binding regions of β-adrenergic receptors amino acid positions covering the putative ligand binding region of TAARs are marked with a plus (+). Highly conserved amino acids of family A GPCRs are marked by a star-symbol (*).
(TIF)

Figure S3 **Differences between amino acids in the putative ligand binding region of human TAARs 2, 5, 6, 8 and 9 compared with hTAAR1.** Amino acids constituting the ligand binding region (side chains as sticks) of hTAAR1 (green) are highlighted at the molecular homology model (backbone, top view). For the hTAAR subtypes 2, 5, 6, 8 and 9 only side chains are shown which are different compared to TAAR1 residues. This comparison reveals that most of the differences are located spatially between TMH3, TMH5 and TMH6 (red translucent circles). In other words, between the interfaces of TMH 2-3-6-7 a region of high similarity for all hTAAR subtypes might exist (green translucent circle).
(TIF)
