# Supplementary figures and images for: Differential Modulation of Beta-Adrenergic Receptor Signaling by Trace Amine-Associated Receptor 1 Agonists
Source: PLoS One. 2011 Oct 31;6(10):e27073. doi: 10.1371/journal.pone.0027073 (PMC3205048; doi:10.1371/journal.pone.0027073)

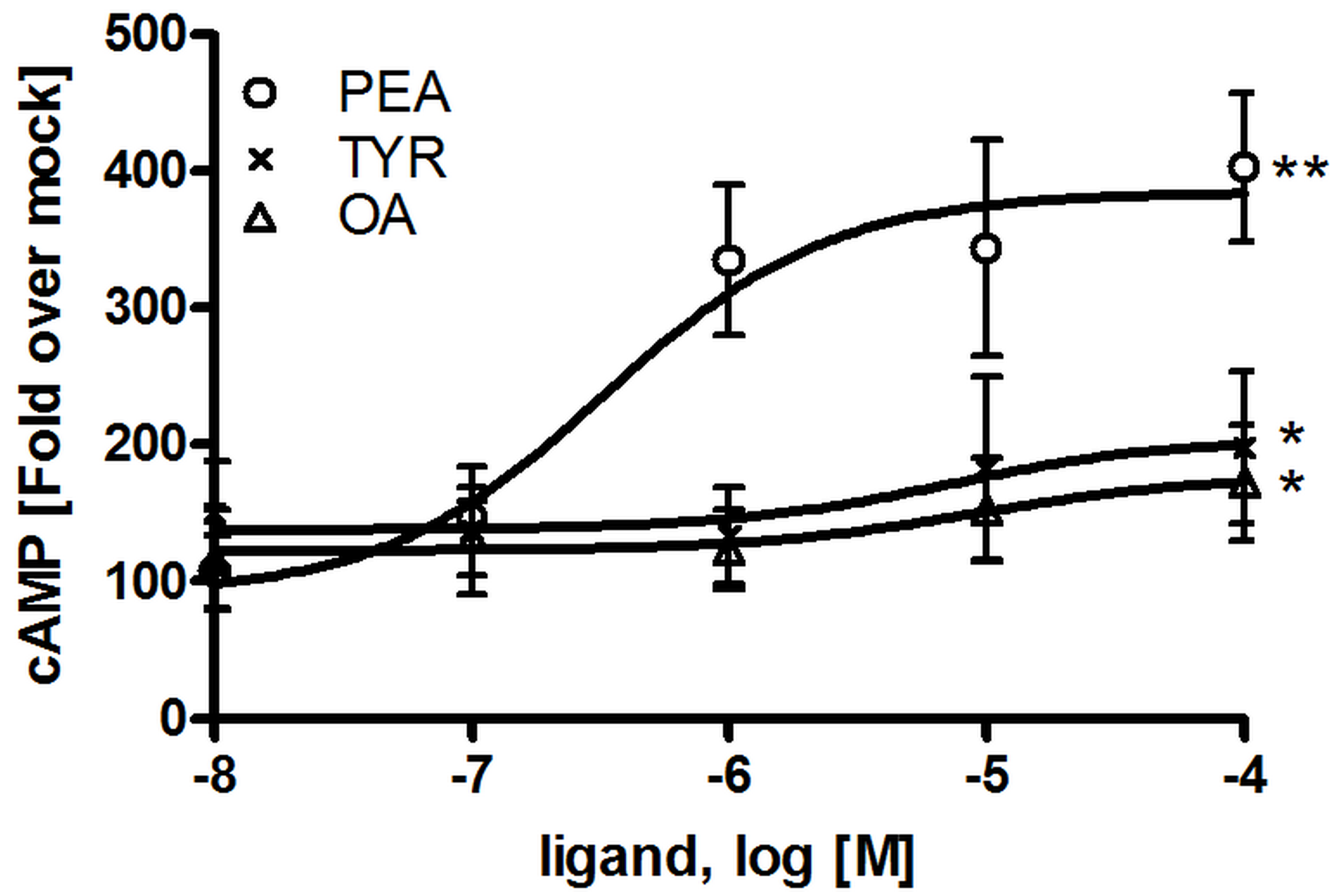

Supplement: Figure S1 — Dose response curves of hTAAR1 agonists. The trace amines tyramine (TYR), beta-phenylethylamine (PEA) and octopamine (OA) activate hTAAR1 via Gs/adenylate cyclase signaling. HEK293 transiently expressing hTAAR1 were stimulated with each trace amine in concentrations ranging 0.1 mM to 10 nM. Shown are dose response curves fold over basal means ± SEM from n≥4 independent experiments of measured cAMP in triplicates as described in Material and Methods. PEA was the most potent agonist (p<0.01), followed by TYR (p<0.05) and OA (p<0.05) for 10 µM each ligand. Data were analyzed using paired one-tailed t-test tested against basal value of hTAAR1. (TIF) [file pone.0027073.s001.tif]

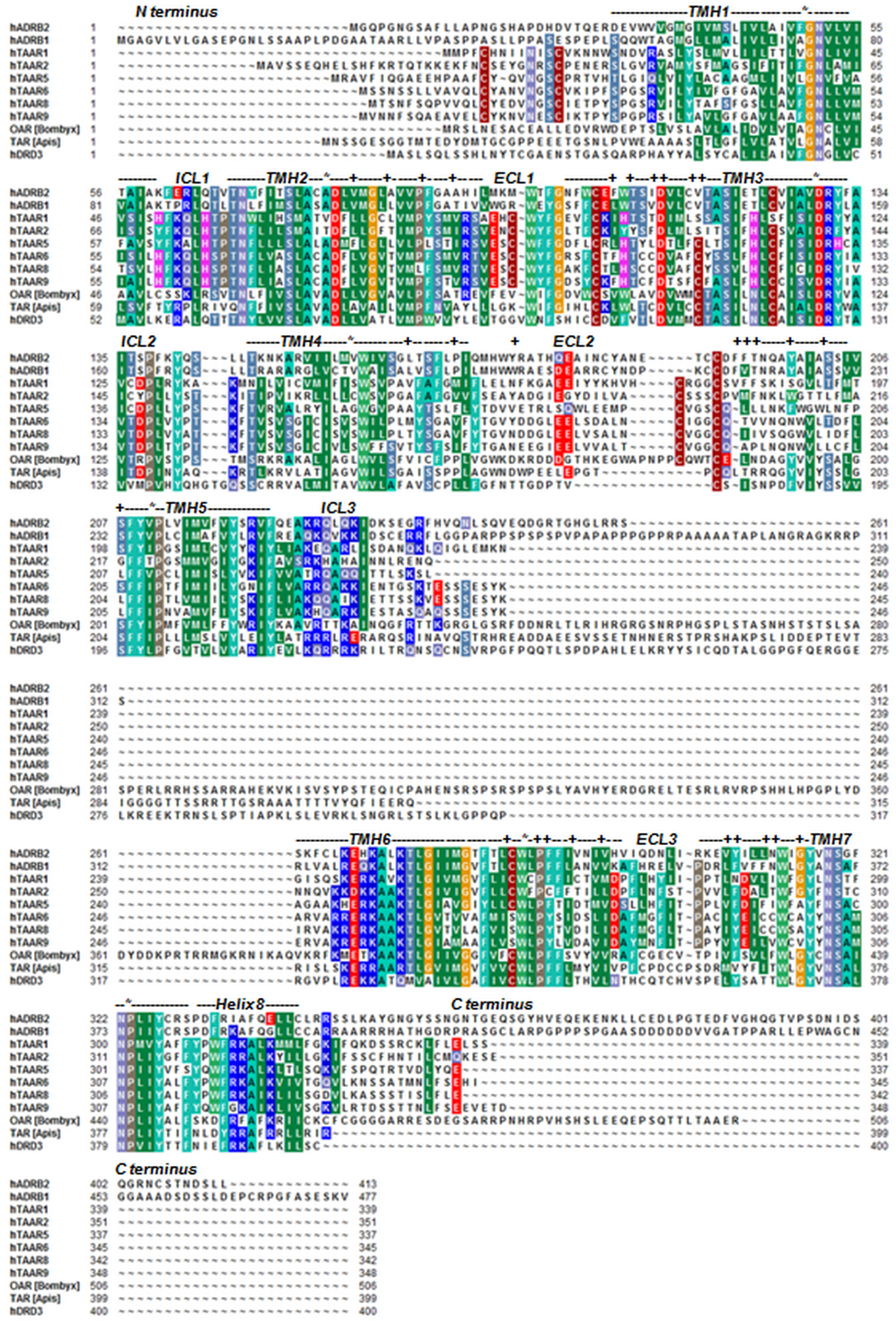

Supplement: Figure S2 — Amino acid sequence alignment of hTAAR1 and homologous receptors. The alignment compares amino acids of ADRB1, ADRB2, human TAAR, invertebrate octopamine (OAR) and tyramine (TAR) receptors and the human dopamine-3 receptor (DRD3). Particular background colors indicating conservation among different receptors and reflecting biophysical properties of the amino acid side chains: black – proline, blue – positively charged, cyan/green – aromatic and hydrophobic, green – hydrophobic, red – negatively charged, gray – hydrophilic, dark-red – cysteines, magenta – histidine. The putative helix dimensions and loop regions are assigned according to observable features in the crystal structure of the inactivated β2-adrenergic receptor (pdb entry code 2RH1). Furthermore, in homology to the ligand binding regions of β-adrenergic receptors amino acid positions covering the putative ligand binding region of TAARs are marked with a plus (+). Highly conserved amino acids of family A GPCRs are marked by a star-symbol (*). (TIF) [file pone.0027073.s002.tif]

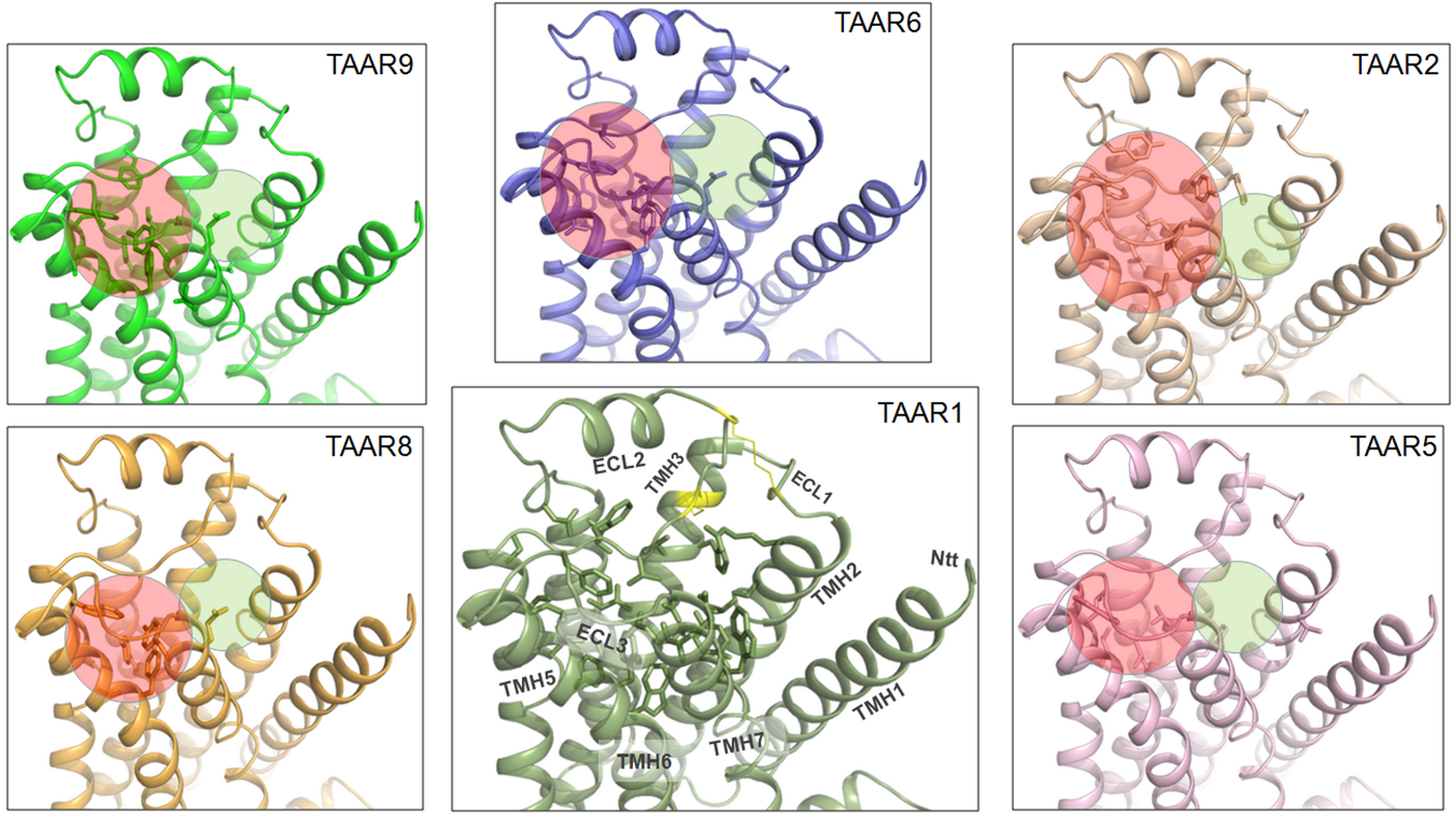

Supplement: Figure S3 — Differences between amino acids in the putative ligand binding region of human TAARs 2, 5, 6, 8 and 9 compared with hTAAR1. Amino acids constituting the ligand binding region (side chains as sticks) of hTAAR1 (green) are highlighted at the molecular homology model (backbone, top view). For the hTAAR subtypes 2, 5, 6, 8 and 9 only side chains are shown which are different compared to TAAR1 residues. This comparison reveals that most of the differences are located spatially between TMH3, TMH5 and TMH6 (red translucent circles). In other words, between the interfaces of TMH 2-3-6-7 a region of high similarity for all hTAAR subtypes might exist (green translucent circle). (TIF) [file pone.0027073.s003.tif]
